# Effects of Autologous Tenocyte Injection for Overuse and Degenerative Tendinopathies: A Systematic Review

**DOI:** 10.3390/jfmk10010095

**Published:** 2025-03-17

**Authors:** Andrea Demeco, Alessandro de Sire, Antonello Salerno, Nicola Marotta, Beatrice Comuni, Matteo Gabbi, Lorenzo Lippi, Marco Invernizzi, Antonio Ammendolia, Cosimo Costantino

**Affiliations:** 1 Department of Medicine and Surgery, University of Parma, 43126 Parma, Italy; andrea.demeco@unipr.it (A.D.); antonello.salerno@unipr.it (A.S.); beatrice.comuni@unipr.it (B.C.); matteo.gabbi@unipr.it (M.G.); cosimo.costantino@unipr.it (C.C.); 2 Physical and Rehabilitative Medicine, Department of Medical and Surgical Sciences, University of Catanzaro “Magna Graecia”, 88100 Catanzaro, Italy; ammendolia@unicz.it; 3 Research Center on Musculoskeletal Health, MusculoSkeletalHealth@UMG, University of Catanzaro “Magna Graecia”, 88100 Catanzaro, Italy; nicola.marotta@unicz.it; 4 Physical and Rehabilitative Medicine, Department of Experimental and Clinical Medicine, University of Catanzaro “Magna Graecia”, 88100 Catanzaro, Italy; 5 Department of Scientific Research, Campus LUdeS Lugano (CH), Off-Campus Semmelweis University of Budapest, 1071 Budapest, Hungary; lorenzolippi.mt@gmail.com; 6 Department of Health Sciences, University of Eastern Piedmont “A. Avogadro”, 28100 Novara, Italy; marco.invernizzi@med.uniupo.it; 7 Translational Medicine, Dipartimento Attività Integrate Ricerca e Innovazione (DAIRI), Azienda Ospedaliera SS. Antonio e Biagio e Cesare Arrigo, 15121 Alessandria, Italy

**Keywords:** rehabilitation, musculoskeletal diseases, regenerative medicine

## Abstract

**Background:** The term tendinopathy commonly describes a series of alterations in the tendon, leading in functional impairment and pain, treated with several approaches, including exercises, physical agents, and injection therapies. Among the latter, autologous tenocyte injection (ATI) involves harvesting tenocytes from a healthy tendon of the patients and then isolating the tenocytes and culturing for 4–5 weeks. To date, there is still a lack of consensus about the efficacy of ATI in improving pain and function; therefore, the present review aimed to assess the role of ATI in the treatment of chronic tendinopathies. **Methods:** Two authors conducted a comprehensive search across PubMed Medline, Web of Science, Scopus, Cochrane Library, and Google Scholar (PROSPERO: CRD42024565211). From 174 articles, we finally included 5 articles. **Results:** The main effect obtained was the pain relief and, consequently, the improvement of patients’ quality of life. The clinical improvement is also evident at MRI in which it is possible to see a progressive reduction with a general disappearance of the T2 signal hyperintensity between 4 months and 1 year. All the articles agree on the safety of ATI in chronic tendinopathies. **Conclusions:** ATI might represent a safe and valuable option in the management of chronic tendinopathies as a second line treatment in the case of resistant tendinopathies, with a minimal risk of side effects.

## 1. Introduction

The term “tendinopathy” commonly describes a series of alterations in the tendon, resulting in reduced functionality and pain. Tendons play an essential role in contributing strength, storing force, and allowing daily activities. During sports, where the load significantly increases, mechanical forces on the tendon also rise, demanding greater effort from the tendon [1,2].

In an “altered” tendon, collagen bundles become highly disorganized, microvascularization increases, and neoinnervation occurs [3,4].

Since 2000, the prevalence of tendinopathies has risen globally, impacting both athletes and people of all generations [5,6,7,8]. The prevalence and incidence of tendinopathies vary across different body parts, influenced by factors such as age, gender, sports type, occupation, and comorbidities. Among lower limbs, the tendinopathies at Achilles tendons (2.4%) and patellar tendons (1.6%) are predominantly affected [9,10]. Moreover, recent studies highlight an increasing incidence of gluteal tendinopathies (4.2%). On the other hand, among upper limb tendinopathies, the rotator cuff tendons are commonly affected (5.5%). Lateral epicondylitis is the prevalent elbow tendinopathy with an incidence of 0.7% [2,5,11,12].

Tendinopathy development involves both modifiable and non-modifiable risk factors [2], e.g., women are more predisposed than men [13]. Furthermore, systemic diseases, such as obesity [14], hypercholesterolemia, and diabetes mellitus [15], impact tendinopathy incidence and patient response to physical therapy [16]. Genetic factors significantly influence tendon homeostasis, affecting the balance between degeneration and repair after injuries [17,18]. Other significant risk factors include restricted or excessive joint movement, muscle deficiency, and impairments in neuromuscular coordination [2].

Athletes commonly receive a diagnosis of tendinopathy, accounting for approximately 30% of all diagnosed injuries [19]. The type of sport and its intensity strongly correlate with the site of tendinopathy onset [20,21,22]; additionally, professional contexts involving high-force or repetitive activities elevate the risk [23,24,25,26].

Tendinopathy begins with repeated functional overload, damaging collagen fibrils. Normally, early lesions trigger a reparative response. However, the tendon’s limited repair capacity, combined with inadequate recovery, leads to progressive matrix damage over time. This results in a gradual loss of structural collagen and additional matrix protein deposition [27,28]. Initially asymptomatic, structural alterations accumulate. Pro-inflammatory cytokines build up, activating nociceptors and causing symptoms [2]. In damaged tendons, tenocytes and immune cells release cytokines (e.g., IL-6, TNF, IL-1β, and IFNγ) and growth factors (PDGF and TGFβ) [29,30,31]. Consequently fibroblasts adopt a pro-inflammatory phenotype [32], leading to increased collagen synthesis (mainly type III collagen) with chaotic fiber arrangement [33].

Furthermore, healthy tendons harbor clusters of stem cells and progenitor cells known as TSPCs. Prolonged inflammatory stimulation leads to TSPCs losing their ability to differentiate into tenocytes. Rather, they promote other cell subtypes (such as osteoblasts, chondrocytes, and adipocytes), further disrupting the typical tendon repair mechanisms [34].

Patients with tendinopathy commonly experience pain and morning stiffness. The onset of tendinopathy represents a vulnerable moment for individuals. Since symptoms may temporarily improve after warming up, individuals often persist with sports, work, or activities. Over time, these symptoms can escalate to constant and debilitating pain during such activities [2].

Beginning with the patient’s reported symptoms, a thorough physical examination is mandatory for diagnosis [35]. Palpation is useful for assessing tendon tenderness, especially for easily palpable tendons and to evaluate other structures such as adipose pads or tendon bursae. Performing pain-provoking tests specific to the affected tendon can be useful [36]: single leg heel raise and hop test in Achilles tendinopathy [35], single leg decline squat and Royal London Hospital test in patellar tendinopathy [37], and resisted wrist/finger extension and gripping an object in elbow tendinopathy (medial or lateral) [38]. Even if tendinopathy diagnosis relies on clinical assessment, imaging (X-ray, MRI, and ultrasonography) can aid in differential diagnosis and ruling out other causes of pain. In particular ultrasound, a widely used imaging method reveals tendon thickening, hypo-echoic regions, disrupted collagen organization, and possible neovascularization [39,40,41,42,43].

Tendinopathies can be treated using various methods, both active and passive. Among the active approaches, tendon load programs are effective as conservative treatment [44,45], including eccentric training, which induces structural adaptation in muscle-tendon units and helps protect tendons from stress and prevent re-injury [2], gradually increasing the load on the tendon to enhance its resistance [46], and isometric exercises to reduce pain [47].

Moreover, instrumental physical therapy, e.g., laser therapy, extra-corporeal shock-wave therapy (ESWT), or injection therapy, is usually employed, while surgical treatment is considered a last resort [48,49,50].

Low-energy laser therapy reduces edema and inflammation, induces analgesia, and supports healing in various musculoskeletal conditions [51].

ESWT, commonly used for calcific rotator cuff tendinopathy, plantar fasciitis, and Achilles tendinopathy [52], employs acoustic waves to enhance soft tissue healing and inhibit pain receptors [53].

Finally, injection therapy has gained ever more importance in the last years and can involve various substances, including hyaluronic acid, platelet-rich plasma (PRP), corticosteroids, and elevated-volume injections [2,11,54,55]. PRP, a centrifuged autologous blood preparation, contains an elevated platelet concentration, either paired with leukocytes or not. Platelet degranulation releases factors such as PDGF, VEGF, IGF1, TGFβ, and EGF, which stimulate tendon healing [56]. Corticosteroid injections are commonly used for tendon pathologies and have a local anti-inflammatory effect, providing pain relief and relaxing muscle spasms [57,58,59,60]. High-volume injections, frequently employed in Achilles tendinopathy, involve the injection of a significant volume of saline solution, often combined with corticosteroids and local anesthetic [61].

In this context, autologous tenocyte injection is a two-stage procedure. In the first stage, a small piece of a healthy tendon is collected under local anesthesia, with or without ultrasound guidance. The tenocytes are then isolated and cultured for 4–5 weeks. In the second phase, the autologous tenocytes obtained from in vitro culture are implanted into the damaged tendon area using ultrasound-guided injections [62,63]. Since these tenocytes are mature homologous cells that maintain their cellular phenotype unchanged, this treatment is considered safe [64].

To date, due to the paucity and heterogeneity of the results of the studies on autologous tenocyte injection in the treatment of tendinopathies, the objective of this systematic review was to investigate the current state of the art and determine the effectiveness of autologous tenocyte injection in improving pain, muscle strength, function, and MRI lesion appearance in patients with overuse and degenerative tendinopathies.

## 2. Materials and Methods

### 2.1. Protocol Design

This systematic review is performed following the guidelines set by PRISMA statement (Preferred Reporting Items for Systematic reviews and Meta-analyses) [65], and it was registered in PROSPERO with number CRD42024565211.

### 2.2. Search Strategy

Two authors independently performed an extensive literature search across several databases, such as PubMed Medline, Scopus, Cochrane Library, and Google Scholar. Their goal was to identify relevant articles published up to April 2024. The research meticulously examined the reference lists of the full-text studies obtained, expert documents, and congress abstracts. The search strategy incorporated the following keywords such as “tendinopathy”, “autologous tenocyte injection”, and “treatment”, along with their synonyms. Depending on the specific database, they employed Boolean operators (“AND”/”OR”) to combine these terms, according to the Cochrane [66]. No publication date filters were applied during the search process. Additionally, a third expert author meticulously reviewed the bibliographic search results and resolved any uncertainties. Table 1 illustrates the search strategy applied.

### 2.3. Study Selection

Following the removal of duplicate articles, 2 authors separately assessed the abstract and the titles of all retrieved references from each database. If either author chose an article during the selection phase, it was thoroughly reviewed by both authors. Any conflicts were resolved by a 3rd author.

We adopted the following PICO (patient/population, intervention, comparison, outcome) model for the study selection: patients with chronic tendinopathy (P); autologous tenocyte injection as treatment (I); no restrictions for the control (C); quantitative and qualitative data related to pain intensity, functional recovery of the affected tendon, and significant morphological changes on imaging as outcomes (O).

Studies were included if they were case series, case reports, randomized controlled trials (RCTs), cohort studies, and case-control studies.

Exclusion criteria were (1) studies conducted on animals; (2) studies not in English; and (3) studies involving patients with recently onset tendinopathies.

### 2.4. Data Extraction

Two researchers separately gathered data from the selected studies using a standardized data collection form in Microsoft Excel. To address any discrepancies, a third author was consulted. We extracted the following information: (1) general study characteristics (such as authors, publication date, nationality, and study design); (2) characteristics of the treatment cohorts (including the number of participants, sex distribution, age range, type of diagnosed tendinopathy, and symptom duration); (3) a description of the intervention type; (4) data related to post-intervention outcomes (evaluated using specific rating scales and imaging techniques); and (5) main findings (including objective variations between pre- and post-intervention scales outcomes, along with mean and standard deviation for each outcome when available).

### 2.5. Outcomes

In this study, we focused on assessing the impact of autologous tenocyte injection therapy in patients with chronic tendinopathies across various anatomical regions. We evaluated several variables: pain, which we assessed verbally or using the visual-analogue scale (VAS); functional capacity recovery, measured with specific evaluation tools based on the location of the tendinopathy; and observable changes in MRI, which served as the reference imaging method.

### 2.6. Quality Assesment

In our current systematic review, we employed the Revised JBI quantitative critical assessment methods to evaluate validity and potential bias. These tools consist of a varying number of questions tailored to the specific study type—for our purposes, case reports and case series [67]. Each question offers four response alternatives: “yes” if the standard was clearly met, “no” if it was not met, “unclear” if it was not fully satisfied, and “not applicable” if the criterion does not apply to the study under examination. Within the tool, certain items address the potential bias, while alternative approaches focus on guaranteeing proper reporting and statistical analysis. A “no” response to any of the questions adversely affects the overall study quality. Importantly, these tools intentionally avoid providing specific cut-off values or scores, allowing for informed inclusion of studies with varying quality in reviews [68].

Two authors separately evaluated the risk of bias and evidence quality, resolving any concerns by consulting with a third researcher. We considered studies with a “yes” number of 6 or more.

## 3. Results

### 3.1. Study Selection

**Figure 1:**
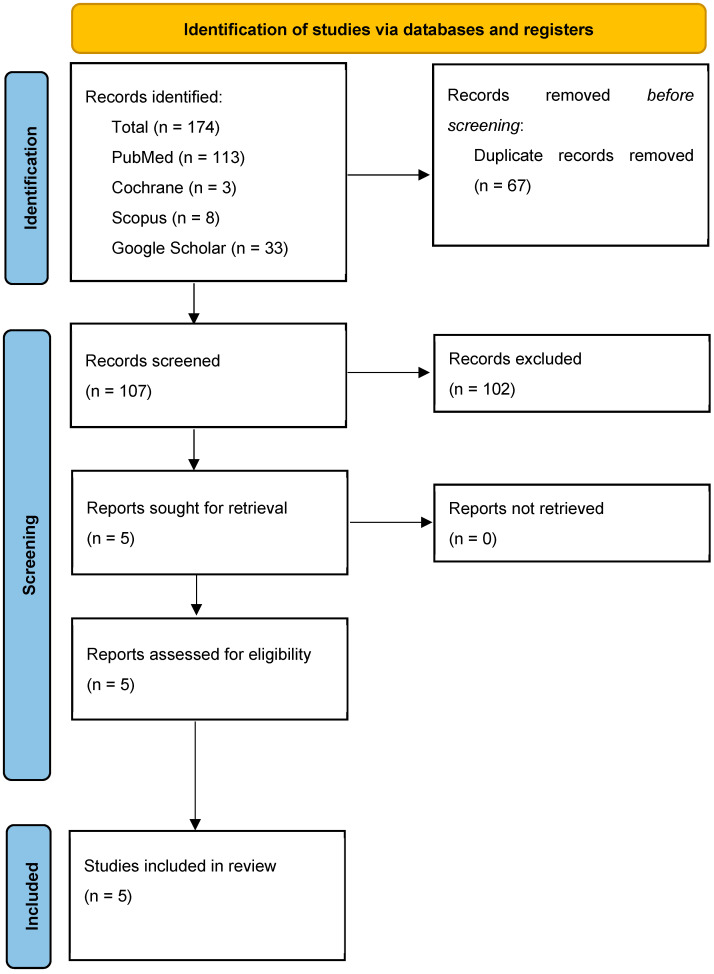
PRISMA flowchart.

The PRISMA flow chart (see Figure 1 for further details) illustrates the article selection procedure. A total of 174 records were obtained from the initial literature search. Sixty-seven studies were eliminated due to duplication, and 107 studies were initially assessed by title and abstract. Eighty-three studies were discarded based on the title/abstract, and 19 studies did not satisfy the inclusion criteria. At least, 5 studies [62,64,69,70,71] were included. Specifically, 3 case series [64,70,71] and 2 case reports [62,69] were included in this review.

### 3.2. Characteristics of the Studies Included in the Review

All the studies featured in this review were carried out in Australia between 2013 and 2018 (2013 [69,70], 2015 [71], 2017 [64], and 2018 [62]). These studies collected data from 50 participants (22 man and 28 woman) aged between 20 and 67, all of whom were affected by various forms of tendinopathy and had ongoing symptoms for at least 6 months. The location of tendinopathy was reported in all selected articles. In particular, two studies considered lateral epicondylitis, one study considered gluteal tendinopathy and two shoulder pain. See Table 2 for further details.

### 3.3. Intervention Protocol

Autologous tenocyte injection varied in terms of tenocyte collection site, tenocyte proliferation time, the number of tenocytes injected, and the type of needle used for injection.

Wang et al. (2013) [69] harvested tenocytes from the subject’s patella tendon under regional anesthesia, and then cellular expansion was performed in a GMP laboratory setting. About 20 days later, 2 mL of tenocyte suspension was administered to the tendon with tendinopathy, guided by ultrasound.

Wang et al. (2013) [70] took tenocytes from patellar tendon. After approximately three weeks, using ultrasound guidance and regional anesthetic, a single injection of autologous tenocytes was performed on patients directly into the tendinopathy site of the extensor carpi radialis brevis tendon.

In Wang et al.’s (2015) [71] study, around three weeks following biopsy, with the aid of an 18-gauge needle and guided by ultrasound, an injection of up to 2 mL of autologous tenocytes, in suspension with 10% autologous human serum, was performed at the tendinopathy site of the extensor carpi radialis brevis tendon.

In Bucher et al.’s [64] study, after about four weeks, a single 2 mL injection of autologous tenocyte suspension was given to the patients. The injection was administered under ultrasound guidance, using a 22-gauge needle, into the area affected by tendinopathy.

Schwab et al. [62] harvested a small section of palmaris longus tendon, which was then cultured. After 7 weeks of tenocyte proliferation, an ultrasound-guided injection of three 1 mL vials containing a total of 5 × 10^6^ tenocytes were performed into the subscapularis tendon of the patients.

### 3.4. Side Effects

No patients experienced adverse effects at the biopsy site. Just three individuals declared slight pain at the tendon biopsy site in Bucher et al.’s study [64], but all these patients showed improvement with topical NSAID gel, and they had no persistent sequelae associated to the intervention site. No contamination, tendon laceration, neurotrauma, hematoma, or calcification was identified at the inoculation site.

Two articles did not evaluate side effects [62,70].

### 3.5. Outcome Measures Assessed

In the reviewed articles, researchers focused on three key outcomes: pain, the function of the injured tendon, and the appearance of tendinopathy on MRI. Four studies used the visual analog scale (VAS) to evaluate pain [64,69,70,71], and the tendon function was evaluated using several validated scales or tools, including the Oxford Shoulder Score [71], QuickDisabilities of the Arm, Shoulder, and Hand (QuickDASH) [69,70,71], Upper Extremity Functional Scale (UEFS) [71], Oxford Hip Score (OHS) [64], and the 36-item Short Form Health Survey (SF-36) [64]. Additionally, grip strength was quantified utilizing the Jamar dynamometer [70,71], and shoulder internal rotation muscular capacity was assessed with a Commander Power Trak II dynamometer [62]. All MRI scans were conducted using a 3 Tesla machine.

### 3.6. Pain

In Wang et al.’s 2013 study [69], they reported a VAS pain score of 1 out of 10 at 10 months after the injection. In Wang et al.’s 2013 study [70], the mean maximum VAS pain score was 5.94 ± 2.24 (median, 5.80). Following autologous tenocyte injection, it increased by 57% at four weeks (*p* < 0.001), 77% at six months (*p* < 0.001), and 86% at 12 months (mean, 0.76; *p* < 0.001). Prior to intervention in Wang et al.’s 2015 study [52], the VAS pain score was 5.94 ± 0.56. At 1-year follow-up, it improved by 86% (0.76 ± 0.14; *p* < 0.001). The final assessment showed a 78% improvement (1.21 ± 0.31), significantly better than the initial state but comparable to the 1-year results (*p* > 0.05).

Bucher et al. [64] observed progress from the starting point to three months post-injection for the VAS (change, −2.8 points; 95% CI, −4.4 to −1.2; *p* = 0.001). The estimated mean improvement in VAS from the starting point to 12 months was −4.1 (95% CI, −2.6 to −5.6; *p* < 0.001). Incremental improvement mainly occurred in the first 3 months. The improvement was sustained at 24 months, with a pain reduction from baseline to 24 months of −4.5 points (95% CI, −6.1 to −2.9; *p* < 0.001).

Schwab et al. [62] reported complete pain remission after 7 weeks from the injection.

### 3.7. Function

Wang et al. 2013 [69] reported a QuickDASH, 13/55 with sports module, 6/20 at 10 months after injection.

In Wang et al.’s 2013 study [70] the mean pre-intervention QuickDASH score was 45.88 ± 15.24. Significant (*p* < 0.001) progress was evident after 4 weeks, with an additional improvement to 12 months, where the mean QuickDASH score decreased to 2.88 ± 0.72 (91% improvement from pre-intervention scores). The grip strength assessment demonstrated a parallel pattern of enhancement.

In Wang et al.’s 2015 study [71], prior to the intervention, the average QuickDASH score was 45.88 ± 3.81. A substantial reduction of 91% was observed at the one-year mark, with the mean score dropping to 3.84 ± 1.05 (*p* < 0.001). At the final assessment, an 84% reduction was maintained, yielding a mean score of 6.61 ± 1.87 (*p* < 0.001). Notably, no statistically significant variation in QuickDASH scores was found between the one-year and final follow-up periods (*p* > 0.05).

The Upper Extremity Functional Scale (UEFS) exhibited a comparable trend. From an initial mean of 31.73 ± 3.75, a 66% enhancement was achieved at one year, resulting in a mean of 9.40 ± 0.52 (*p* < 0.001). This improvement was largely sustained at the final follow-up, showing a 64% enhancement with a mean of 9.20 ± 0.39 (*p* < 0.001 compared to baseline). Again, no significant difference was detected between the one-year and final follow-up UEFS scores.

Grip strength measurements displayed a progressive increase. From a baseline of 19.85 ± 2.81 kg, a significant improvement of 132.6% was recorded at the one-year follow-up, with the mean reaching 37.38 ± 3.30 kg (*p* < 0.001). This upward trend continued, culminating in a 208% improvement at the final follow-up, where the mean grip strength was 46.60 ± 3.46 kg (*p* < 0.001).

In Bucher et al.’s study [64], statistical analysis revealed significant gains in OHS from the pre-injection starting point to the 6-month follow-up (change, 8.3 points; 95% CI, 3.9–12.8; *p* = 0.009). A clinically important improvement of at least 11 points was observed in seven out of the twelve patients. The observed improvements in the OHS score were not found to be associated with patient age (Spearman rho, −0.306; *p* = 0.334) or symptom duration (Spearman rho, −0.182; *p* = 0.572).

Schwab et al. [62] also demonstrated a progressive increase in hand grip strength from the pre-autologous tenocyte injection value at three and seven weeks after the injection even though, following the return to full training, strength results did not fully recover to baseline.

### 3.8. MRI

Following autologous tenocyte injections, Wang et al. (2013) [69] conducted 3 Tesla MRI evaluations at both 4 and 10 months. Two experienced musculoskeletal radiologists independently assessed rotator cuff tendinopathy, partial tear thickness, and anteroposterior (AP) tear size. The study revealed that while tendinopathy, characterized by tendon thickening and persistent focal signal increase, was mitigated at the 4-month mark, this positive change did not sustain through the 10-month follow-up. In contrast, the partial-thickness rim-rent tear was not observed at either time point, indicating a successful healing process.

Wang et al., in 2013 [70], utilized magnetic resonance imaging to evaluate the severity of tendinopathy and tears at the insertion of the common extensor tendon before and after a 12-month treatment period. The mean MRI score, starting at 4.31 ± 1.14 (scale 2–6), significantly improved to 2.88 ± 0.72 (*p* < 0.001) after 12 months. The study demonstrated significant correlations between MRI scores and clinical outcomes, including QuickDASH (R = 0.545, *p* = 0.001), maximum pain (R = 0.448, *p* = 0.010), and grip strength (R = −0.494, *p* = 0.004). All patients diagnosed with grade-3 tendinosis showed a reduction in their scores following treatment. Of the sixteen patients with grade-3 tears, four experienced a decrease in tear severity, two remained unchanged, and one grade-2 tear worsened to grade 3. One patient, deemed a non-responder at 3 months, elected to undergo surgery for lateral epicondylitis and subsequently withdrew from the study.

In Wang et al. [71] in 2015, the MRI was utilized to determine the severity of tendinopathy and the size of the tear at the insertion of the common extensor tendon. Initially, the average MRI score was recorded at 4.31, with a standard deviation of 0.28. After a year, a notable reduction to 2.88 ± 0.18 was observed (*p* < 0.001), and this improvement persisted for five years, averaging 2.87 ± 0.19 (*p* < 0.001). Most participants, specifically all but two, exhibited consistent MRI scores between the one-year and final assessments. In one case, the tendinopathy component of the score showed further enhancement at 4.3 years post-intervention, moving from 2 at one year to 1, resulting in an overall improvement from 3 to 2. Conversely, another patient experienced a minor increase in the tear score, from 1 to 2, due to a minimal partial-thickness tear in the deep extensor carpi radialis brevis tendon, though this was less pronounced than pre-treatment. Interestingly, this patient continued to engage in tennis.

In the study conducted by Bucher et al. [64], the researchers aimed to determine if MRI-detected tendinopathy characteristics were associated with clinical outcomes following autologous tenocyte injections. Their analysis revealed no notable changes in tendon features, as assessed by MRI, between pre- and post-injection evaluations. The consistency of MRI measurements, as determined by intrarater agreement, was generally strong, with PABAK values ranging from 0.727 to 1.00, except for the signal intensity in the lateral gluteus medius tendon.

In Schwab et al. [62], three radiologists, working independently and blinded, confirmed a marked post-assessment change in tendon appearance on MRI. The majority, specifically two of the three, judged the tear to be entirely healed, and all observed an improvement in tendinopathy.

### 3.9. Other Outcomes

Bucher et al. [64] utilized the SF-36 questionnaire to assess overall health, generating both mental (MCS) and physical (PCS) component scores. The PCS subscale showed a trend of progressive enhancement, with the most significant gains occurring within the initial three months post-intervention. Prior to the intervention, the average PCS score was 28.1 (SD 8.5), ranging from 17.4 to 46.1. At 12 months, the estimated average increase in the PCS was 15.2 points (95% CI 9.8–20.5; *p* < 0.001). This positive change in PCS scores was sustained over a 24-month period, with an estimated average improvement of 12.8 points (95% CI 7.3–18.3; *p* < 0.001) from baseline.

Wang et al. (2015) [71] and Bucher et al. [64] used a 7-point scale to evaluate patient satisfaction following treatment.

In Wang et al.’s 2015 study [71], at final follow-up, a significant majority, 93% (n = 14), of patients reported high or moderate satisfaction with their autologous tenocyte injection treatment. Only one patient, whose tear worsened, expressed uncertainty, assigning a score of 5. Furthermore, a considerable number of patients (10 out of 15) resumed activities known to trigger pain in lateral epicondylitis, such as darts, tennis, weight lifting, and gardening.

Bucher et al. [64] reported that all 12 patients completed a satisfaction survey 12 months after their surgery. Of those, five expressed strong satisfaction, three reported being satisfied or quite satisfied, two were undecided, and two were dissatisfied with their results. Notably, patient satisfaction at 12 months closely aligned with the degree of improvement observed at the same time point; those experiencing the greatest improvement were highly satisfied, while those with minimal improvement were either dissatisfied or unsure.

### 3.10. Study Limitations

The two main limitations present in all the articles considered were the number of patients and the absence of a control group. The study design did not incorporate either a comparative alternative treatment or a placebo, precluding a definitive assessment of the treatment’s superiority or the potential influence of a placebo effect on the results [62,64,69,70,71].

Another limitation found in Bucher et al.’s study [64] is the utilization of the OHS, a clinical scale specific for hip osteoarthritis, as the main clinical outcome measure. Additionally, they explored changes detectable by MRI that correlated with clinical improvement. However, they found that clear radiological improvement was not present in the majority of cases despite the reported clinical improvement. This could be a limitation due to the lack of a validated tool to assess MRI improvement following an intervention, or it could be due to the post-injection MRI being performed too early.

### 3.11. Study Quality

We used the revised Joanna Briggs Institute (JBI) quantitative critical appraisal tools to evaluate validity and risk of bias. In particular, the two case reports scored 7/8 [62,69] (see Table 3).

## 4. Discussion

The therapeutic landscape in tendinopathies is vast and not always effective due to the wide heterogeneity of patients and the complexity of lesions and repair mechanisms underlying the pathology [57,72,73].

This review aimed to investigate the security and therapeutic effect of autologous tenocyte treatment in tendinopathies.

Autologous tenocyte implantation is a minimally invasive technique and almost always well tolerated by patients who show an early reduction in symptoms, followed by a functional recovery. This is particularly useful to allow an early rehabilitation plan [74,75].

In particular, all studies examined the safety of autologous tenocyte injection, and, throughout the follow-up period, no patient reported significant adverse effects or immunological reactions. Furthermore, a clear majority of participants showed a high degree of satisfaction after the treatment, demonstrating that not only was the treatment well tolerated but was also subjectively perceived as effective.

All areas examined, namely, pain, the recovery of function, and changes in lesion imaging, showed an improvement starting from the first month of follow-up. In all studies, the improvement continued for the first 6 months and then remained substantially unchanged up to one year, regardless of the location of the tendinopathy. However, since no control group was present in any of the studies considered, it cannot be stated with certainty that this improvement was actually due to the tenocyte injection. Nevertheless, this is an encouraging result, also considering that recent studies have shown that PRP, currently the most widely used conservative approach in clinical practice to promote tendon regeneration, has an efficacy that does not appear to be better to that of placebo in alleviating discomfort or improving tendon function [76].

However, the only study in this review that extended the follow-up time to 4.5 years [71] showed a general worsening of pain symptoms and a progressive decrease in the function of the injured tendon, although both of mild entity, without, however, returning to pre-treatment levels. Autologous tenocyte injection shows a very similar trend to corticosteroid injections but with better results and fewer side effects.

Corticosteroid treatment in insertional tendinopathies, in fact, has efficacy primarily in the short term. In the long term, progressive worsening occurs, with high rates of recurrence [77,78,79]. Furthermore, although corticosteroid injection induces a reduction in pain and inflammation and an enhanced sonographic visualization of the tendon, it has adverse effects ranging from a deficit in the strength of the injured tendon to atrophy or rupture of the same, in 82% of clinical trials [80].

Despite these problems highlighted in corticosteroid injections, some studies have shown that image-guided high-volume injections (HVIGIs) of normal saline, local anesthetic, and corticosteroid determine good results regarding pain relief and the enhancement of functional activity in patients with different types of tendinopathy. Furthermore, Maffulli et al. have highlighted positive effects also in HVIGI with normal saline, local anesthetic, and aprotinin, therefore, without the use of corticosteroids. This technique proves to be promising, with results similar to those of autologous tenocyte injection [53].

As for the evaluation of pain, specifically in the group of patients suffering from lateral epicondylitis, the studies considered have shown that, beyond the first year, it ceased to improve, remaining virtually unchanged at average VAS values of 1.2.

These data can be compared with that of the method commonly used as a last resort in chronic tendinopathies. A 2024 review [81] was analyzed that compares open, percutaneous, and arthroscopic surgery in lateral epicondylitis. From this, it can be seen that the improvement in pain is extremely variable both between the various interventions and between people undergoing the same type of intervention; however, average VAS values of 1.2 represent the lowest values among all three surgical techniques [72,82]. With autologous tenocyte injection, unlike surgical techniques, being a conservative treatment, such an outcome is strongly encouraging [83,84].

As is known, on imaging, tendinopathies classically appear with a thickening of the tendon associated with an increase in signal in T2 sequences, while concomitant tears, complete or partial, have a variable appearance: they generally present with a hyperintensity of the signal in T2 sequences, but, in the more advanced stages, the deposition of scar tissue can hide this hyperintensity of signal [85,86]. Both patients in the two case reports examined [62,69] at the MRI control of their respective lesions showed a progressive reduction in the tear in parallel with a reduction in the aforementioned signs of tendinopathy, respectively, 4 and 6 months after the tenocyte injection.

It is evident that the injection at the site of the tear not only did not determine its enlargement but, on the contrary, triggered a process of healing and repair. In Schwab et al. [62], these data are particularly significant as the patient had already undergone corticosteroid injections and, 7 weeks before the injection, an arthroscopic evaluation with PRP injection in the same site without, however, obtaining any benefit concerning pain reduction or the recovery of performance. However, it is essential to consider that, in this case, it is not possible to determine if the patient’s clinical improvement is attributable only to autologous tenocyte injection or to the combination of this method with previous therapeutic interventions.

Another aspect of this review to consider is the cost: autologous tenocyte injection, involving a laboratory phase, is certainly burdened by higher costs and longer waiting times compared to other therapeutic strategies.

This review is not free of limitations. Firstly, the included studies exhibited a heterogeneous patient population, varying in age, clinical condition, and tendinopathy site. Notably, the precise clinical condition of patients was not consistently detailed across all studies, which could significantly influence healing times. Moreover, the lack of uniformity in evaluation scales across studies, coupled with variations in intervention methods and protocols, hindered a fully objective and direct comparison of findings.

## 5. Conclusions

Collectively, the results of this systematic review evidenced that autologous tenocyte injection could represent a valuable option in the management of chronic tendinopathies, also considering its safety, with a minimal risk of side effects. The main effect obtained was the pain relief and, consequently, the improvement of patients’ quality of life. The clinical improvement is also evident at MRI in which it is possible to see a progressive reduction with a general disappearance of the T2 signal hyperintensity between 4 months and 1 year. However, even if the costs are higher than corticosteroid injection or PRP, autologous tenocyte injection can be considered as a second line treatment in the case of resistant tendinopathies. However, more research is necessary to confirm the efficiency of this treatment. In particular, studies conducted in other countries are necessary to obtain more globally representative data. The inclusion of a control group is essential to ensure a comparison with both untreated patients and patients treated with other injection therapies. Comparing patients with tendinopathy in the same location would be very useful so that the same evaluation scales can always be used to facilitate direct data comparability.

## Figures and Tables

**Table 1:** Search strategy.

PUBMED:
(autologous tenocyte injection OR autologous tenocyte implant) AND (tendon OR tendinopathy) AND treatment
SCOPUS:
(TITLE-ABS ((autologous tenocyte injection OR autologous tenocyte implant) AND (tendon OR tendinopathy) AND treatment)
WEB OF SCIENCE:
(autologous tenocyte injection OR autologous tenocyte implant) AND (tendon OR tendinopathy) AND treatment

**Table 2:** Main characteristics of the included studies.

Article	Nation	Population	Intervention	Outcomes	Results
Wang et al., 2013 [69]	Australia	n = 1; Age: 20; Diagnosis: Increased tendon size associated with a partial-thickness supraspinatus rim-rent tear with fluid signal; Symptom duration: 3 y	Tenocytes were obtained from the patient’s patellar tendon. Three weeks later, 2 mL of tenocyte suspension was administered under ultrasound guidance.	At 10 months: VAS pain, Oxford Shoulder Score, and QuickDASH with sports module). At four and ten months post-ATI, control with 3 Tesla MRIs.	At 4 and 10 months, the athlete reported no pain, and the partial-thickness rim-rent tear had healed, becoming undetectable.
Wang et al., 2013 [70]	Australia	n = 20 (11M/9F); Age: 49.4 ± 7.69, range, 37–63; Diagnosis: refractory lateral epicondylitis; Symptom duration: ≥6 months	Patients received a single injection of autologous tenocytes at the extensor carpi radialis brevis tendon under ultrasound guidance and local anesthetic.	VAS for pain, quick Disabilities of the Arm, Shoulder, and Hand (QuickDASH), and grip strength measurements before and after ATI at one, two, three, six, and twelve months. At 12 months, these were also evaluated via MRI.	ATI treatment resulted in significant improvements across pain, function, strength, and MRI findings within 12 months:
Wang et al., 2015 [71]	Australia	n = 16 (9M/7F); Age: 47.76 ± 1.79 years, range, 37–63 years; Diagnosis: chronic lateral epicondylitis; Symptom duration: 29.24 ± 14.26 (6–240) mo; Follow-up duration: 4.06 ± 0.33 (0.5–5) y.	2 mL of autologous tenocytes was injected into the extensor carpi radialis brevis under ultrasound guidance.	QuickDASH, UEFS, VAS, and dynamometer. Patient satisfaction was evaluated on a scale of 0 to 10 at final follow-up. The MRI assessment was performed at T0 and after twelve months.	ATI treatment demonstrated significant improvements in pain, function, strength, and MRI findings, with high patient satisfaction.
Bucher et al., 2017 [64]	Australia	n = 12 (0M/12F); Age: mean 52.6 y (range, 41–67 y); Diagnosis: symptomatic gluteal tendinopathy; Symptom duration: 33 m (range, 6–144 m).	Single tenocyte injection was injected into the tendinopathic area, under ultrasound guidance.	OHS, VAS, SF-36, and patient satisfaction (evaluated on a scale of 0 to 7). All outcomes were evaluated at T0 and after three, six, twelve, and twenty-four months.	Autologous tenocyte injection resulted in significant clinical improvements in pain, function, and physical health over 12–24 months but did not show significant changes in tendon features on MRI.
Schwab et al., 2018 [62]	Australia	n = 1 (M); Age: 28 y; Diagnosis: anterior/lateral shoulder pain surrounding the deltoid tubercle; Symptom duration: 2 months	Three 1 mL vials containin g a total of 5 × 10^6 ^tenocytes were injected into the subscapularis tendon under ultrasound guidance.	MRI images after seven and eighteen months	An MRI showed a marked decrease in tear size and an improvement in tendon morphology. Futhermore, athletes returned to complete training.

Abbreviations: VAS, visual analog scale; QuickDASH, quick Disabilities of the Arm, Shoulder, and Hand; ATI, autologous tenocyte injection; OHS, Oxford Hip Score; IR, internal rotation; HHD, hand-held dynamometer; MCS, mental component score; PCS, physical component score; UEFS, upper extremity functional score; SF-36, 36-item Short Form Health Survey.

**Table 3:** Revised JBI quantitative critical appraisal tools to assess validity and risk of bias for case report.

	Wang, 2013 [69]	Schwab, 2018 [62]
Were patient’s demographic characteristics clearly described?	Y	Y
Was the patient’s history clearly described and presented as a timeline?	U	Y
Was the current clinical condition of the patient on presentation clearly described?	Y	Y
Were diagnostic tests or assessment methods and the results clearly described?	Y	Y
Was the intervention(s) or treatment procedure(s) clearly described?	Y	Y
Was the post-intervention clinical condition clearly described?	Y	Y
Were adverse events (harms) or unanticipated events identified and described?	N	N
Does the case report provide takeaway lessons?	Y	Y
Inclusion?	Y	Y

The three case series scored 8/10 [64,69,71] (see Table 4).

**Table 4:** Revised JBI quantitative critical appraisal tools to assess validity and risk of bias for case series.

	Wang, 2013 [69]	Wang, 2015 [71]	Bucher, 2017 [64]
Were there clear criteria for inclusion in the case series?	Y	Y	Y
Was the condition measured in a standard, reliable way for all participants included in the case series?	Y	Y	Y
Were valid methods used for identification of the condition for all participants included in the case series?	Y	Y	Y
Did the case series have consecutive inclusion of participants?	N	N	Y
Did the case series have complete inclusion of participants?	U	U	Y
Was there clear reporting of the demographics of the participants in the study?	Y	Y	N
Was there clear reporting of clinical information of the participants?	U	U	U
Were the outcomes or follow-up results of cases clearly reported?	Y	Y	Y
Was there clear reporting of the presenting site(s)/clinic(s) demographic information?	Y	Y	Y
Was statistical analysis appropriate?	Y	Y	Y
Inclusion?	Y	Y	Y

## Data Availability

The dataset is available on reasonable request from the corresponding author.
